# Treatment of pyoderma gangrenosum with apremilast monotherapy

**DOI:** 10.1016/j.jdcr.2022.10.001

**Published:** 2022-10-11

**Authors:** Zachary A. Bordeaux, Shawn G. Kwatra, Cameron E. West

**Affiliations:** a Department of Dermatology, Johns Hopkins University School of Medicine, Baltimore, Maryland; b Department of Oncology, Johns Hopkins University School of Medicine, Baltimore, Maryland; c Genzada Pharmaceuticals, Hutchinson, Kansas; d US Dermatology Partners, Wichita, Kansas

**Keywords:** case reports, cytokines, immunomodulators, neutrophils/neutrophilic, pyoderma gangrenosum, therapeutics, PG, Pyoderma gangrenosum, TNF, Tumor necrosis factor, PDE4, phosphodiesterase 4, IL, Interleukin, cAMP, cyclic adenosine monophosphate

## Introduction

Pyoderma gangrenosum (PG) is a rare inflammatory neutrophilic dermatosis that is characterized by rapidly developing painful ulcers with overhanging borders and peripheral erythema.^1^ It is typically treated with local and systemic immunomodulatory agents such as corticosteroids, cyclosporin, dapsone, and tumor necrosis factor (TNF)-α inhibitors,^2^ but many lesions exhibit treatment-resistant or relapse following a successful therapeutic response.^1^^,^^2^ Additionally, as there is currently a paucity of clinical data to guide the treatment of PG, management often revolves around guidelines set forth by case reports or small clinical trials,^1^^,^^2^ highlighting the need to identify novel therapeutic strategies for this disease. Here, we report a case of PG treated with apremilast monotherapy, which is a phosphodiesterase 4 (PDE4) inhibitor.

## Case report

**Fig 1:**
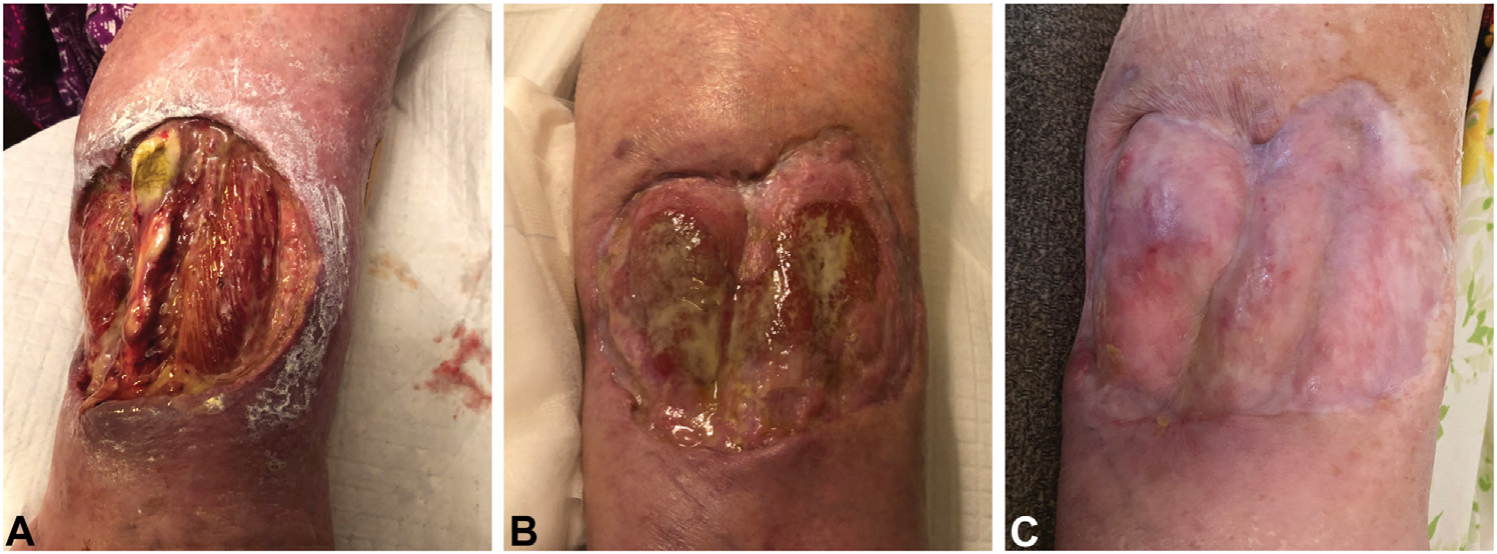
Pyoderma gangrenosum before and after treatment. **A,** Patient’s leg before treatment. **B,** Patients leg after 4 months of apremilast monotherapy. **C,** Patient’s leg after 3 years of apremilast monotherapy.

A 68-year-old female with a 5-year history of PG of the right lower extremity presented for evaluation of her worsening skin condition. Physical exam showed a full-thickness non-healing ulcer with an undermined border measuring 12 cm in diameter that extended to the underlying musculature (Fig 1, *A*). The patient reported severe 8 out of 10 pain score on a numeric pain rating scale that prevented ambulation, causing her to be wheelchair-bound. She also carried a medical history of obesity, type II diabetes mellitus, major depressive disorder, and mixed iron/vitamin B_12_ deficiency anemia.

The patient had not seen a dermatologist since her initial PG diagnosis 5 years ago. Over that time period, she was treated with surgical debridement, oral and topical corticosteroids, and wound care. She initially improved on oral glucocorticoids but discontinued due to worsening glycemic control. Surgical debridement, wound care, and topical triamcinolone resulted in only minimal improvement.

Upon presentation to dermatology, the patient declined a biopsy and was treated for PG empirically. She wished to avoid injection agents, and the presence of hematologic comorbidities made TNF-α inhibitors and dapsone poor therapeutic options. Hematologic workup was negative for myelodysplasia and hematologic malignancy. The patient declined referral to gastroenterology, though extensive history was not suggestive of underlying inflammatory bowel disease. Due to these factors, apremilast monotherapy was initiated at a dose of 30 mg twice daily. Within 4 months, her lesion had decreased to 8 cm in diameter (Fig 1, *B*). After 3 years of apremilast therapy, her PG had almost entirely cleared with some residual scarring (Fig 1, *C*). She now rated her pain score as 0 out of 10 and regained the ability to ambulate. Interestingly, she reported the common adverse effects of diarrhea and decreased appetite, and lost 40 pounds over the course of treatment. Eventually, her weight stabilized, her appetite returned, and her glycemic control improved. The patient remains on apremilast 30 mg orally twice daily and has had no recurrences to date (3 months after complete resolution).

## Discussion

The pathogenesis of PG remains poorly understood but is thought to involve local and systemic immunologic dysregulation resulting in a neutrophil-dominant skin infiltration.^1^^,^^2^ Specifically, elevated levels of TNF-α, interleukin (IL)-1β, IL-6, IL-8, IL-17, and IL-23 have been identified in the lesions of PG and are thought to play a key role in its progression.^3^ TNF-α, IL-8, IL-17, and IL-23 all directly or indirectly promote neutrophil migration, and their upregulation in PG likely contributes to the neutrophilic infiltration seen in this disease.^1^

Previous case reports describe successful treatment of PG with apremilast in combination with oral glucocorticoids and subcutaneous methotrexate,^4^ and with infliximab.^5^ However, we report successful therapeutic response to apremilast monotherapy. PDE4 is the major enzyme class responsible for hydrolysis of cyclic adenosine monophosphate. Inhibition of PDE4 results in increased levels of intracellular cyclic adenosine monophosphate, which modulates the inflammatory response of immune and non-immune cells to decrease the production of TNF-α, IL-2, IL-8, IL-12, and L-23.^5^ The suppression of neutrophil attracting cytokines and TNF-α may explain the observed improvement of PG with apremilast.

This study is limited by the lack of histopathologic confirmation of PG. However, other considerations, such as keratinocyte carcinomas and infectious etiologies, were excluded. The initial favorable clinical response to oral corticosteroids, as well as positive pathergy, also militate in favor of PG. Additionally, while the initial response to apremilast was favorable, the protracted timeframe for complete resolution may suggest spontaneous resolution. Though this is plausible, the disease course had been progressive until intervention with apremilast, and although all healed completely, additional lesions continued to develop at sites of minor trauma during treatment, supporting the fact that the clinical response was likely due to apremilast rather than spontaneous healing. Finally, the protracted response may also be explained by delayed healing common in diabetic patients and the anatomic location on the lower leg. It is worth noting, however, that prior case reports utilizing apremilast in conjunction with other medications noted a clinical response in a shorter timeframe. One study reported marked improvement within 2 weeks,^6^ and the other noted near complete resolution of PG after 5 months of treatment.^4^ However, the ulceration in these studies was less extensive than that of the patient in this report, and it is thus reasonable to expect that resolution may take additional time. Additionally, although complete healing took nearly 3 years in the present case, improvements were seen as early as 4 months which is similar to the timeframe reported by Laird et al^4^ In conclusion, given the lack of clinical data to guide the treatment of PG,^1^ this report may provide further rationale for the study of apremilast in the treatment of this condition.

## Conflicts of interest

Dr Kwatra is an advisory board member/consultant for Abbvie, Galderma, Incyte Corporation, Pfizer Inc, Regeneron Pharmaceuticals, and Kiniksa Pharmaceuticals and has received grant funding from Galderma, Pfizer Inc, and Kiniksa Pharmaceuticals. Dr West is a director and officer at Genzada Pharmaceuticals. Mr Bordeaux has no conflicts of interest to declare.
